# Pancreatic Elastase Affects Liver Injury by Activating Pro-inflammatory Cytokines in Kupffer Cells *via* the JAK2/STAT3 Signaling Pathway

**DOI:** 10.2174/0115665240363959250624052201

**Published:** 2025-07-01

**Authors:** Ying Feng, Xinxin Jin, Haoyu Xu, Bo Sun, Meixia Guo, Minli Li

**Affiliations:** 1 Department of Gastroenterology, Jinling Hospital, Nanjing University, School of Medicine, Nanjing, Jiangsu 210002, P.R. China;; 2 Department of Gastroenterology, Nanjing Yimin Hospital, Nanjing, Jiangsu 210002, P.R. China

**Keywords:** JAK/STAT pathway, Kupffer cells, liver injury, acute pancreatitis, proinflammatory cytokines, pancreatic elastase

## Abstract

**Introduction:**

This study aimed to investigate the role of JAK2 (Janus kinase 2)/STAT3 (signal transducer and activator of transcription 3) signaling in liver injury during severe acute pancreatitis (SAP), focusing on pancreatic elastase- and lipopolysaccharide (LPS)-induced Kupffer cell (KC) activation.

**Methods:**

A rat SAP model was established via retrograde taurocholic acid infusion into the biliopancreatic duct. Inflammatory cytokine levels and JAK2/STAT3 pathway activity were quantified in liver tissues. KCs were treated with elastase/LPS ± AG490 (JAK2 inhibitor). Proinflammatory cytokines, RNA, and protein expression were analyzed.

**Results and Discussion:**

SAP rats exhibited elevated TNF-α, IL-6, and IL-18 levels in both serum and liver tissues, with JAK2/STAT3 pathway activation. AG490 administration suppressed JAK2/STAT3 activation, reduced inflammation, and alleviated liver injury. Similarly, KCs treated with elastase and LPS showed increased proinflammatory cytokine levels and JAK2/STAT3 upregulation, which were mitigated by AG490 treatment.

**Conclusion:**

The findings highlighted the pivotal role of the JAK2/STAT3 signaling pathway in SAP-induced liver injury. Selective inhibition of this pathway by AG490 could reduce inflammation and protect against liver damage, suggesting its potential as a therapeutic target for inflammatory liver diseases.

## 1 INTRODUCTION

Acute pancreatitis (AP) has a high global incidence, with severe acute pancreatitis (SAP) accounting for over 20% of cases [1]. Furthermore, SAP frequently triggers a systemic inflammatory response, leading to fatal multiple organ dysfunction, with hepatic injury frequently being a common complication. Research indicates that during the early stages of SAP, excessive inflammatory cytokine release into the liver plays a pivotal role in the development of liver injury [2, 3]. Additionally, studies demonstrate that the early inflammatory response can induce edema and substantial necrosis [4]. However, the precise triggering events and molecular pathophysiological mechanisms remain inadequately explored. Consequently, understanding the regulatory mechanisms of inflammatory pathways in SAP is essential for identifying novel therapeutic targets [5].

The JAK2/STAT3 signaling pathway represents a critical inflammatory mechanism and constitutes the primary pathway involved in the initiation of inflammatory activity [6, 7]. Cytokines activate JAK2 signaling through membrane-proximal domain receptors and subsequently trigger phosphorylation events [8]. Activated JAK2 recruits STAT3 via SH2 domain-containing molecules near phosphorylated sites [9]. This signaling cascade is strongly associated with various inflammatory disorders. In ulcerative colitis, abnormal JAK2/STAT3 pathway activation contributes to intestinal inflammation, oxidative damage, and apoptotic cell death [10]. Similarly, alpinetin mitigates neuroinflammation by inhibiting the JAK2/STAT3 pathway in microglia [11]. Notably, JAK2/STAT3 activation has been implicated in pancreatitis progression through systemic inflammatory responses [12, 13]. Kupffer cells (KCs), the liver's resident macrophages, play a central role in hepatic inflammatory responses during SAP [14]. During SAP progression, pancreatic inflammatory cytokines migrate to liver sinusoids, where KCs amplify inflammation through cytokine release, exacerbating liver injury. Despite this established role, the mechanisms sustaining KC-mediated inflammation and JAK2/STAT3 pathway regulation in organ injury remain poorly understood. This study investigated the JAK2/STAT3 signaling pathway's role in SAP-associated liver injury, utilizing both *in vivo* models and *in vitro* KC cultures stimulated with pancreatic elastase and lipopolysaccharide (LPS).

## 2 MATERIALS AND METHODS

### 2.1 Animals

Male Sprague-Dawley rats, aged 8 to 10 weeks and weighing between 200 to 250 grams, were obtained from the Animal Experimentation Center of Jinling Hospital [license number SCXK (Su) 2018-00^17^]. All experimental procedures adhered to international guidelines for the ethical treatment and handling of laboratory animals.

### 2.2 *In vivo* Experimental Design

A total of twenty-four rats were randomly assigned to three groups (n = 8 per group): (1) control group (0.9% saline injection), (2) SAP group (4% sodium taurocholate infusion), and (3) SAP-AG490 group (AG490 pretreatment). The SAP group received continuous retrograde infusion of 4% sodium taurocholate (0.1 mg/100 g body weight; Sigma‒Aldrich, cat. no. S0900000) into the biliopancreatic duct at 20 μl/min using a micropump. The SAP-AG490 group received AG490 (8 mg/kg; Santa Cruz Biotechnology, cat. no. sc-202046) dissolved in DMSO (30 nmol/ml) via the inferior vena cava 30 minutes prior to taurocholate infusion. Animals were euthanized 18 hours post-intervention. Blood samples were collected via right atrial puncture, allowed to clot for 15 minutes at room temperature, and centrifuged at 3,000 rpm for 10 minutes. Serum was stored at -80°C for subsequent analysis. Pancreatic and liver tissues were either fixed for histology/immunofluorescence or frozen at -80°C for western blotting.

### 2.3 KC Extraction and Culture

Healthy male rats were anesthetized via inhalation, and their abdomens were surgically opened. LPS and intravenous collagenase were sequentially injected into the portal vein. The liver was excised once it exhibited a yellow coloration and soft consistency. The harvested liver was digested with collagenase, and nonparenchymal cells were isolated from parenchymal cells using differential centrifugation. KCs were further purified from nonparenchymal cells via Percoll density gradient centrifugation. Adherent KCs were selected by cultivating the cells in Dulbecco’s modified Eagle medium (DMEM) for 4 hours, which was followed by removal of non-adherent cells. KC viability (>95%) was confirmed using trypan blue exclusion and ink phagocytosis assays.

Purified KCs were seeded into two 24-well culture plates at a density of 2×10^6 cells per well and incubated for 48 hours. Cells were randomly divided into seven experimental groups as follows: control: saline (30 μl/ml); LPS: LPS (50 ng/ml; Sigma‒Aldrich, cat. no. SMB00610); elastase: pancreatic elastase (1 U/ml; Sigma‒Aldrich, cat. no. E7885); elastase + LPS: LPS (50 ng/ml) + elastase (1 U/ml); AG490 + LPS: AG490 (30 nmol/ml; Sigma) + LPS (50 ng/ml); AG490 + elastase: AG490 (30 nmol/ml) + elastase (1 U/ml); AG490 + LPS + elastase: AG490 (30 nmol/ml) + LPS (50 ng/ml) + elastase (1 U/ml). Cells were pretreated with AG490 (where applicable) for 2 hours before specific treatments. Supernatants and cells were collected 24 hours post-treatment for analysis. Each experiment included eight biological replicates, with three technical replicates per treatment condition.

### 2.4 Serological Assays

Serum amylase (AMY), alanine aminotransferase (ALT), and aspartate aminotransferase (AST) levels were measured using kits provided by Beijing Leadman Biochemistry Technology Co., Ltd., China, following the manufacturer’s protocol.

### 2.5 HE Staining of Pancreatic and Liver Tissues

A trained pathologist stained the pancreatic and liver tissues with hematoxylin-eosin (H&E). The severity of severe acute pancreatitis (SAP) was then analyzed through histological assessment of these tissues under optical microscopy, focusing on indicators, such as edema, inflammation, hemorrhage, and necrosis. The pancreatic histological scoring was based on the criteria established by Schmidt *et al.* [15]. Scoring was carried out considering the four aspects: edema, inflammatory infiltration, hemorrhage, and necrosis, with a scale of 0-4 points for each aspect, and the total score ranged from 0 to 16 points. The hepatic histological scoring referred to the criteria of Camargo *et al.* [16]. Scoring was conducted for disorganization of hepatic lobular structure, inflammatory infiltration, hepatic sinusoid stenosis, and hepatocyte vacuolar degeneration, with a scale of 0-3 points for each item, and the total score ranged from 0 to 12 points. All scores were independently evaluated blindly by two pathologists, and the average value was taken to reduce subjective bias.

### 2.6 Measurement of TNF-α, IL-6, and IL-18 Levels *Via* ELISA

The concentrations of cytokines, namely TNF-α (cat. no. RTA00), IL-6 (cat. no. CATR6000B), and IL-18 (cat. no. DY992; all R&D Systems Europe, Ltd.), were quantified in both serum and supernatant samples using ELISA kits. All procedures adhered strictly to the manufacturer's guidelines.

### 2.7 Reverse Transcription‑quantitative PCR (RT-qPCR)

Total RNA was extracted from KCs utilizing TRIzol reagent (cat. no. 15596-018; Invitrogen; Thermo Fisher Scientific, Inc.), following the manufacturer's prescribed protocols. Subsequently, 1 microgram of total RNA underwent reverse transcription employing a cDNA synthesis kit (cat. no. AR0063; Wuhan Boster Biological Technology, Ltd.). cDNA was amplified via qPCR analysis using the QuantStudio 7 Pro Real-Time PCR platform (Applied Biosystems, Foster City, USA) with the following reaction mixture: 5μL cDNA, 0.5μL each of forward and reverse primers, 5μL 5×PCR buffer, 0.124μL Taq DNA polymerase, and 20μL distilled water. The thermal cycling protocol entailed an initial denaturation step at 95°C for 5 minutes, followed by 40 cycles consisting of denaturation at 95°C for 30 seconds, primer annealing at 55°C for 30 seconds, and extension at 72°C for 1 minute. The expression levels of RNA were determined using the 2^-ΔΔCt^ method, with β-actin serving as the internal reference gene. All experiments included three technical replicates. Primer sequences (Sangon Biotech Co., Ltd., China) are listed in Table **1**.

### 2.8 Liver Immunohistochemistry and KC Immunofluorescence

Rat liver tissues were fixed, embedded, and sectioned into 5-μm-thick slices. After deparaffinization and antigen retrieval in citrate buffer (pH 6.0), sections were incubated overnight at 4°C with the following primary antibodies: rabbit anti-rat JAK2 (1:200 dilution; Cell Signaling Technology, cat. no. 3230) and STAT3 (1:200 dilution; Cell Signaling Technology, cat. no. 30835). Sections were washed three times with PBS and incubated with a fluorescein isothiocyanate (FITC)-conjugated goat anti-rabbit secondary antibody (1:200 dilution; Wuhan Boster Biological Technology, cat. no. BA1010) for 15 minutes at room temperature (25°C). Protein expression was quantified using Image-Pro Plus 6.0 software (Media Cybernetics, USA) at 200× magnification.

KCs were fixed in 4% paraformaldehyde and PBS for 30 minutes at 25°C, permeabilized with 0.1% Triton X-100 (in PBS) for 10 minutes, and blocked with 3% bovine serum albumin (BSA) containing 0.2% Tween 20 for 30 minutes. Cells were incubated overnight at 4°C with rabbit anti-rat JAK2 (10μg/ml; Abcam, cat. no. ab108596) and STAT3 (10μg/ml; Cell Signaling Technology, cat. no. 30835). After PBS washes, cells were stained with FITC-conjugated goat anti-rabbit IgG (1:100 dilution; Sigma, cat. no. AP307F) for 1 hour at 25°C. Coverslips were mounted and visualized using an Olympus Bx51 fluorescence microscope (Olympus, USA).

### 2.9 Western Blot Analysis

Total protein was extracted from liver tissues and Kupffer cells (KCs) using RIPA lysis buffer (Beyotime Biotechnology, China) containing protease inhibitors. Protein concentration was measured using a BCA assay kit (Thermo Fisher Scientific, USA). Proteins (30 μg per lane) were separated via 10% SDS-PAGE and transferred to PVDF membranes. Membranes were blocked with 5% non-fat milk for 1 hour at 25°C and incubated overnight at 4°C with the following primary antibodies: JAK2 (1:1,000 dilution; Abcam, cat. no. ab108596) and STAT3 (1:1,000 dilution; Cell Signaling Technology, cat. no. 30835). After washing, membranes were incubated with HRP-conjugated goat anti-rabbit IgG (1:5,000 dilution; Beyotime Biotechnology, cat. no. A0208) for 1 hour at 25°C. Signals were detected using an ECL substrate (Bio-Rad, USA) and quantified using ImageJ software (NIH, USA).

### 2.10 Statistical Analysis

Statistical analyses were performed using IBM SPSS Statistics 26.0 (IBM Corp., Armonk, NY, USA), while graphical representations were crafted with GraphPad Prism version 9.3.1 (GraphPad Software, San Diego, CA, USA). Continuous variables were reported as mean ± standard deviation (SD). The Kolmogorov–Smirnov test was employed to verify normal distribution. Normally distributed data were analyzed by one-way ANOVA with Tukey's multiple-comparison test, while non-normally distributed data were assessed using the Kruskal-Wallis test with Dunn's correction for pairwise comparisons. Statistical significance was set at *P*<0.05.

## 3 RESULTS

### 3.1 AG490 Alleviated Acute Pancreatitis and Liver Injury

The SAP group showed significantly elevated serum levels of AMY, ALT, and AST compared to the control group (*P* < 0.05). Furthermore, treatment with AG-490 mitigated the taurocholic acid-induced elevation of these liver enzymes (*P* < 0.05; Table **2**).

**Fig. (1):**
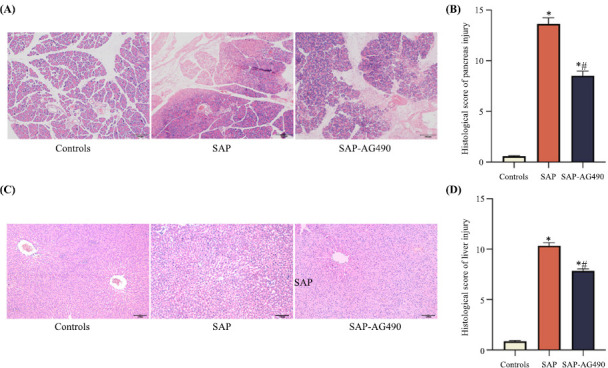
Pathological injury of the pancreas and liver in rats. (**A**) HE staining results of the pancreas in each group (magnification, ×200). (**B**) Histological score of pancreas injury. (**C**) HE staining results of livers from each group (magnification, ×200). (**D**) Histological score of liver injury. *P < 0.05 *vs*. the control group; #*P* < 0.05 *vs*. the SAP group.

Histological analysis via H&E staining revealed normal pancreatic and hepatic architecture in controls. In the SAP group, pancreatic tissues displayed severe acinar cell edema, inflammatory infiltration, focal hemorrhage, and necrosis, while liver tissues exhibited disorganized hepatic lobules, inflammatory cell infiltration, narrowed hepatic sinusoids, hepatocyte vacuolar degeneration, and nuclear pyknosis. The SAP-AG490 group showed milder histological damage compared to the SAP group (*P* < 0.05; Fig. **1A**, **C**). Quantitative histopathology scores are shown in Fig. (**1B**, **D**).

### 3.2 AG490 Suppressed the Expression of the JAK2/STAT3 Signaling Pathway in the Liver and Exhibited an Anti-inflammatory Effect

**Fig. (2):**
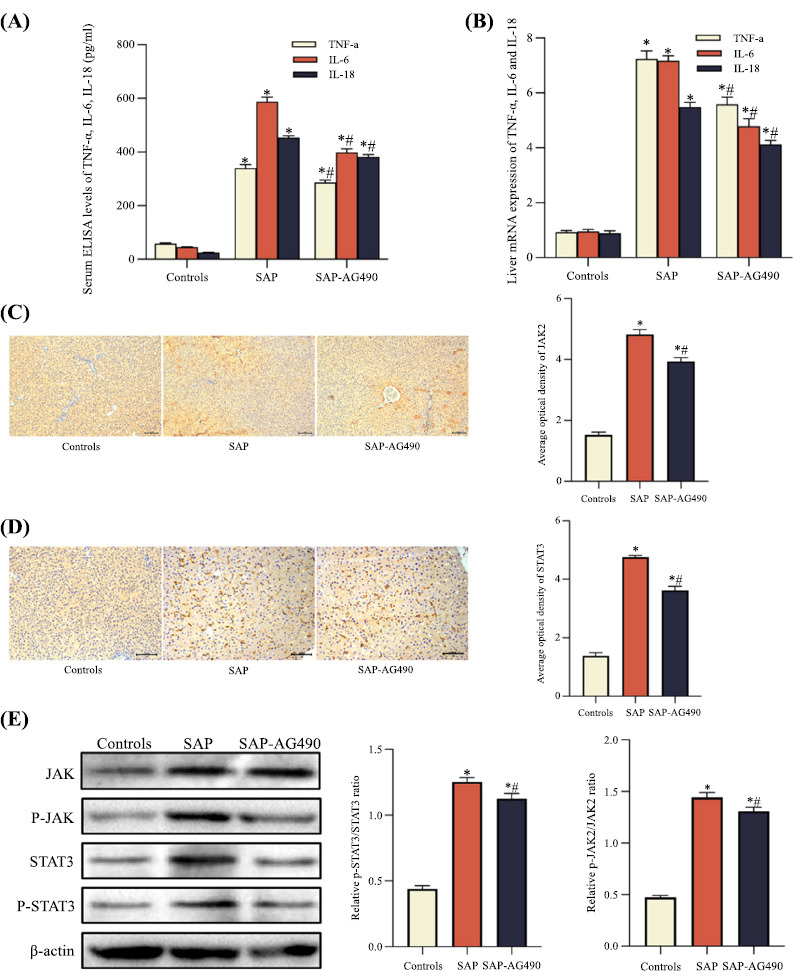
AG490 inhibited the expression of the JAK2/STAT3 pathway in the liver and played an anti-inflammatory role. (**A**) Serum ELISA levels of TNF-α, IL-6, and IL-18 in different groups. (**B**) mRNA expression of TNF-α, IL-6 and IL-18 in liver tissues. (**C**) Immunohistochemical staining of JAK2 in liver tissues (magnification, ×200). (**D**) Immunohistochemical staining of STAT3 in liver tissues (magnification, ×200). (**E**) Western blot analysis of JAK2, p-JAK2, STAT3, and p-STAT3 proteins in the liver. *P < 0.05 *vs*. the control group; #*P* < 0.05 *vs*. the SAP group.

Inflammatory cytokines play a pivotal role in both local and systemic inflammatory responses during SAP. To investigate the mechanisms underlying the protective effects of AG490, we quantified the expression levels of TNF-α, IL-6, and IL-18 in serum (Fig. **2A**) and liver tissue samples (Fig. **2B**) using ELISA and RT-qPCR. Compared to the control group, the SAP group exhibited significantly elevated levels of these inflammatory cytokines (*P*<0.05). Notably, pretreatment with AG490 significantly reduced the SAP-induced increase in cytokine levels (*P* < 0.05).

Immunohistochemical analysis indicated minimal expression of JAK2 and STAT3 in the liver tissues of the control group. In contrast, the SAP group showed activation of the JAK2/STAT3 pathway, with a marked upregulation of JAK2 expression in the cytoplasm (Fig. **2C**) and STAT3 expression in the nucleus (Fig. **2D**). AG490, a JAK2 inhibitor, significantly reduced JAK2 and STAT3 expression (*P* < 0.05), as confirmed by Western blot analysis (Fig. **2E**). Furthermore, AG490 pretreatment mitigated the SAP-induced upregulation of JAK2, STAT3, and their phosphorylated forms (*P* < 0.05).

### 3.3 AG490 Inhibited KCs Activation and Downregulated the JAK2/STAT3 Pathway *In vitro*

**Fig. (3):**
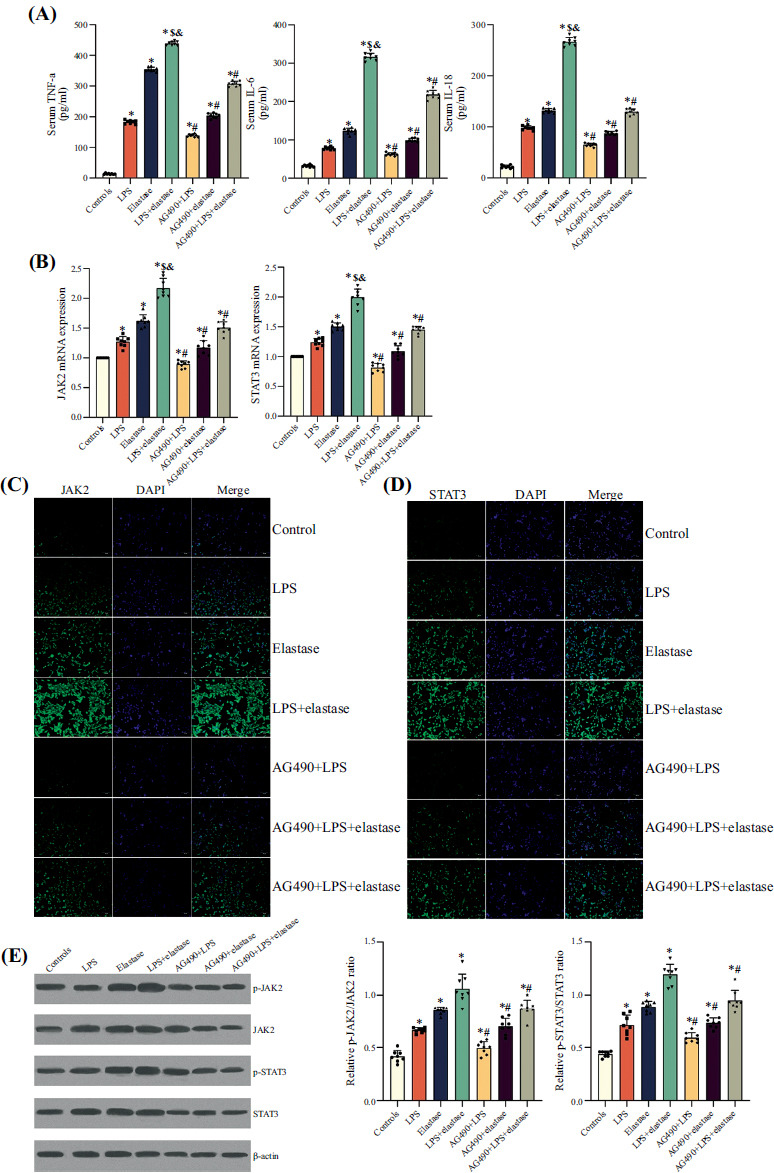
Anti-inflammatory and inhibitory effects of AG490 on the JAK2/STAT3 pathway *in vitro*. (**A**) The levels of TNF-α, IL-6, and IL-18 in the supernatant were detected via ELISA. (**B**) mRNA expression of JAK2 and STAT3 in KCs. (**C**) IF staining of JAK2 in Kupffer cells (original magnification, 200×). (**D**) IF staining of STAT3 in Kupffer cells (original magnification, 200×). (**E**) Western blot analysis of the protein expression of JAK2, p-JAK2, STAT3, and p-STAT3 in KCs from different groups. *P < 0.05 *vs*. the control group; $*P*<0.05 *vs*. the LPS group; &*P*<0.05 *vs*. the elastase group; #*P* < 0.05 *vs*. the SAP group.

Stimulation with LPS and pancreatic elastase induced a marked increase in the secretion of proinflammatory cytokines (TNF-α, IL-6, IL-18) from cultured KCs. Cytokine levels were significantly higher in the elastase-treated group compared to the LPS-treated group (*P*<0.05), with the combination-treated group (LPS + elastase) showing the highest levels. AG490 pretreatment significantly attenuated this cytokine surge (Fig. **3A**, *P*<0.05).

JAK2 and STAT3 mRNA expressions were upregulated in KCs following LPS/elastase treatment. Elastase induced greater mRNA upregulation than LPS alone (*P*<0.05), while the combination treatment resulted in the most pronounced effect. AG490 suppressed both JAK2 and STAT3 mRNA levels (*P*<0.05; Fig. **3B**). Immunofluorescence staining confirmed these findings; control KCs exhibited minimal JAK2/STAT3 signal, whereas LPS/elastase treatment dramatically increased expression, which was reversed by AG490 (Fig. **3C**, **D**).

Western blot analysis revealed elevated phosphorylated JAK2 (p-JAK2) and phosphorylated STAT3 (p-STAT3) levels in LPS/elastase-treated KCs, confirming pathway activation. AG490 pretreatment abolished this phosphorylation (*P* < 0.05; Fig. **3E**).

The results demonstrated that AG490 inhibited KC activation by suppressing JAK2/STAT3 pathway activation, confirming its anti-inflammatory efficacy *in vitro*.

## 4 DISCUSSION

SAP is a globally prevalent gastrointestinal disorder with rising incidence and mortality rates [17]. The release of digestive enzymes into the pancreatic tissue, combined with a systemic inflammatory cascade, plays a key role in pancreatitis progression and multi-organ dysfunction [18-20]. Specifically, liver damage represents a grave outcome of SAP, exacerbating systemic inflammation and elevating the mortality risk [21]. Despite its clinical significance, the molecular mechanisms driving SAP-related liver injury remain poorly understood. KCs, the liver’s resident macrophages, are central mediators of hepatic inflammation during SAP [22, 23]. Moreover, these cells are primary producers of proinflammatory cytokines (TNF-α, IL-6, IL-18) [24-26], which propagate systemic organ failure. Critically, KCs’ activation directly contributes to pancreatitis-associated liver injury by releasing cytokines that exacerbate hepatocellular damage [14, 27]. Thus, targeting KC-driven cytokine signaling is pivotal for mitigating liver injury in SAP. While prior studies implicate pancreatic elastase in KC activation and highlight the JAK2/STAT3 pathway in SAP progression [8, 18, 28], the precise mechanism linking elastase-induced JAK2/STAT3 signaling to acute liver injury remains unresolved.

The JAK2/STAT3 signaling pathway is a central regulator of the inflammatory response. Activated by cytokines, such as IL-6, this pathway amplifies inflam-mation via transcriptional regulation of proinflammatory genes. Cytokine binding triggers JAK2-mediated phosphorylation of STAT3, which dimerizes and translocates to the nucleus to regulate genes involved in apoptosis, autophagy, and immune modulation. While JAK2/STAT3 is extensively studied in inflammatory and neoplastic diseases, its role in SAP-associated liver injury is emerging. Clinically approved JAK inhibitors (e.g., tofacitinib) ameliorate immune-mediated liver damage [29], and our prior work demonstrated JAK2/STAT3-mediated inflammation in SAP-related hepatic injury [30]. Numerous studies have indicated that the JAK/STAT signaling pathway may be closely related to the proinflammatory differentiation of macrophages [31]. In macrophages, JAK2/STAT3 signaling drives proinflammatory polarization by activating STAT subtypes [32, 33]. Notably, the JAK2 inhibitor AG490 suppressed LPS/IFN-γ-induced HMGB1 and IL-6 secretion in macrophages [34]. However, the mechanistic link between KC-specific JAK2/STAT3 activation and SAP-associated liver injury remains uncharacterized.

In this study, the SAP group showed markedly elevated serum AMY, ALT, and AST levels, with histological evidence of pancreatic necrosis, hepatic inflammatory infiltration, and hemorrhage compared to controls. SAP triggered a systemic inflammatory response, characterized by increased serum TNF-α, IL-6, and IL-18 levels and rapid activation of hepatic JAK2/STAT3 signaling. Western blot confirmed elevated JAK2, p-JAK2, STAT3, and p-STAT3 expressions in SAP liver tissues, indicating pathway hyperactivity driving liver injury. Supporting our findings, Li *et al.* demonstrated that JAK2/STAT3 inhibition reversed hepatic damage in ischemia-reperfusion models [35]. Crucially, AG490 pretreatment suppressed JAK2 activation, reduced STAT3 phosphorylation, and lowered proinflammatory cytokine levels (*p* < 0.05), confirming its therapeutic potential for SAP-associated liver injury. While JAK2/STAT3 is central to SAP pathology, NF-κB, MAPK, and NLRP3 inflammasome pathways may interact to amplify liver injury. NF-κB drives TNF-α/IL-6 production in response to pancreatic elastase [3, 36], while MAPK signaling mediates oxidative stress and hepatocyte apoptosis [37, 38]. NLRP3 activation by DAMPs promotes IL-1β/IL-18 maturation, intensifying hepatic inflammation [4, 39]. Notably, STAT3-NLRP3 crosstalk (via transcriptional regulation) [40] suggests a feedforward loop between these pathways. Future studies should explore multi-target interventions blocking JAK2/STAT3 while modulating NF-κB, MAPK, or NLRP3 to disrupt this inflammatory network.

Elastase has been utilized in numerous models to imitate the symptoms of pancreatitis and induce macrophages in the liver to produce proinflammatory cytokines [41-43]. In our study, elastase/LPS-treated KCs recapitulated key features of SAP-induced liver injury, demonstrating JAK2/STAT3 pathway activation as a critical mediator. LPS-induced cytokine release from KCs triggers systemic inflammation [36], making it an essential component for modeling SAP-associated liver injury *in vitro*.

Elevated elastase levels correlated strongly with systemic complications in SAP patients [39]. Our findings directly linked elastase to KC-driven TNF-α, IL-6, and IL-18 secretion, mirroring clinical observations of cytokine-mediated organ failure [44]. These results support the paradigm that SAP complications arise from systemic cytokine storms rather than localized pancreatic inflammation alone.

JAK and STAT activation is widespread in inflammatory cells throughout the pancreas and liver during SAP [38]. KCs express IL-6R and IFN-γR, which initiate JAK2/STAT3 signaling upon cytokine binding [45]. In this study, elastase treatment rapidly induced JAK2 phosphorylation in KCs, reaching peak activation within 12 hours, followed by robust STAT3 activation. Similarly, the JAK2 inhibitor AG490 clearly suppressed JAK2 phosphorylation activation in elastase-stimulated Kupffer cells. Moreover, AG490 notably decreased STAT3 phosphorylation in Kupffer cells, underscoring the importance of JAK2 upstream in this response. Additionally, AG490 suppressed JAK2 production by diminishing elastase-triggered proinflammatory cytokine production and inhibiting STAT3 activation in Kupffer cells. Thus, the induction of the JAK2/STAT3 pathway by elastase in Kupffer cells is dependent on JAK2 and involves cytoplasmic STAT3 phosphorylation and nuclear translocation.

Compelling evidence implicates NF-κB, a master regulator of inflammation, in SAP pathogenesis, with elevated expression observed in pancreatic and hepatic tissues [37, 46]. NF-κB synergizes with proinflammatory cytokines (TNF-α, IL-6) to drive pancreatitis-associated liver injury. We quantified KC-secreted inflammatory mediators and their systemic effects, finding that LPS robustly upregulated TNF-α, IL-6, and IL-18 in KCs. Mechanistically, LPS activates TLR4-dependent signaling, inducing downstream effectors (NF-κB, MAPK, IRF3) that trigger cytokine production [47]. Rapid JAK2/STAT3 activation occurred post-LPS stimulation, establishing this pathway as critical for proinflammatory gene expression. Elastase effectively modeled pancreatitis by inducing KC-derived cytokine storms, while combined elastase/LPS treatment caused marked elevation of TNF-α, IL-6, and IL-18 versus controls. Sustained upregulation of JAK2/STAT3 in treated KCs correlated with progressive inflammation. Notably, cytokine overexpression amplified JAK2/STAT3 levels, while IL-6-induced JAK2 phosphorylation revealed autocrine regulation within KCs. Crucially, elastase/LPS triggered rapid JAK2/STAT3 activation, highlighting this pathway as a central mediator in SAP progression.

**Fig. (4):**
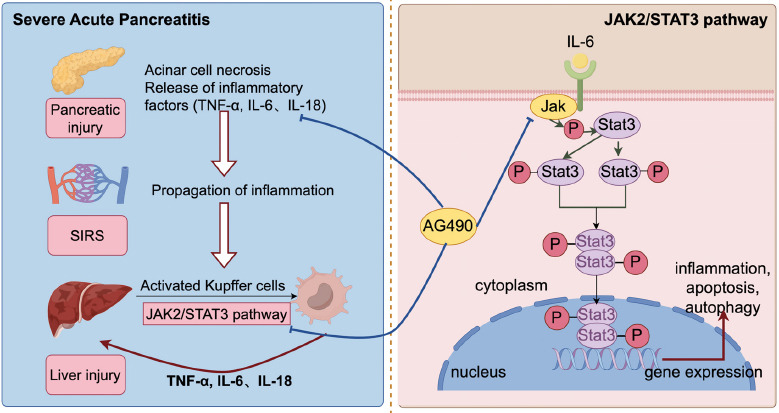
Proposed mechanism of AG490 in SAP-associated liver injury.

The JAK2 inhibitor AG490 suppressed inflammatory signaling by downstream JAK2 activation and blocking STAT3 phosphorylation [48]. In the ICU-AW model, AG490 demonstrated the ability to block the IL-6-induced activation of the JAK2/STAT3 signaling pathway, leading to decreased STAT3 phosphorylation and mitigation of sepsis-associated muscle atrophy [49]. AG490 selectively inhibited JAK, thereby contributing to the reduction in the production of inflammatory cytokines, including IL-1β, IL-6, and TNF-α, in hepatocyte cell lines stimulated by LPS [50]. While AG490's hepatic effects remain underexplored, our study demonstrated that elastase/LPS-activated JAK2/ STAT3 promoted TNF-α/IL-6/IL-18 production in KCs. AG490 pretreatment abolished JAK2 phosphorylation and STAT3 nuclear translocation, confirming its JAK2-specific inhibition. This mechanistic link between JAK2/STAT3 activity and KC-mediated inflammation validates JAK2/STAT3 inhibition as a therapeutic strategy for SAP-associated liver injury. Our proposed model (Fig. **4**) illustrated AG490's dual protective mechanism: (1) pancreatic JAK2/STAT3 suppression reduced initial macrophage activation; (2) hepatic STAT3 inhibition in KCs decreased proinflammatory cytokine release (TNF-α, IL-6, IL-18) and prevented endothelial damage. This dual action prevented systemic inflammation and multiorgan failure.

AG490 inhibited JAK2 phosphorylation (p-JAK2) and subsequent STAT3 nuclear translocation in Kupffer cells (KCs), thereby suppressing the release of pro-inflammatory cytokines (TNF-α, IL-6, IL-18). This dual action attenuated pancreatic necrosis and hepatic sinusoidal damage, breaking the cycle of systemic inflammation in SAP. Red arrows indicate activation pathways; blue blunted lines indicate AG490-mediated inhibition.

## STUDY LIMITATIONS

Our study has involved several limitations. First, the rodent SAP model could not fully recapitulate human SAP complexity due to species-specific differences in disease progression, challenging clinical translatability. Second, the modest sample size limited statistical power to detect smaller effect sizes; large-scale vali-dation studies are thus warranted. Third, while focusing on JAK2/STAT3, we did not explore contributions from NF-κB and NLRP3 pathways, leaving pathway cross-talk unaddressed. Lastly, potential off-target effects of AG490 were not comprehensively characterized. Notwithstanding these constraints, our findings offered critical insights into JAK2/STAT3's role in SAP-associated liver injury and established a framework for developing pathway-targeted therapies.

## CONCLUSION

In summary, our study demonstrated that SAP induces liver injury through the activation of the JAK2/STAT3 signaling pathway in KCs, leading to the release of pro-inflammatory cytokines, such as TNF-α, IL-6, and IL-18. The use of JAK2 inhibitor AG490 significantly alleviated liver injury by downregulating the JAK2/STAT3 pathway, reducing inflammation, and protecting against liver damage. The findings highlighted the crucial role of JAK2/STAT3 signaling pathway in the pathophysiology of AP-associated liver injury and suggested that targeting this pathway may offer a promising therapeutic strategy for managing inflammatory liver diseases associated with SAP. Future research should focus on further elucidating the molecular mechanisms underlying this pathway's involvement in SAP and exploring its potential as a therapeutic target in clinical settings.

## Figures and Tables

**Table 1:** Sequences of primers used in RT-PCR.

**RNA**	**Sequences of primers**
TNF-α	Forward, 5′-CGAGTGACAAGCCCGTAGCC-3′, Reverse, 5′-GGATGAACACGCCAGTCGCC-3′
IL-6	Forward, 5´-ATGAACAGCGATGATGCACTG-3´, Reverse, 5´-CATATTGCCAGTTCTTCGTAG-3´
IL-18	Forward, 5´-GAACCAGTAGAAGACAATTGC-3´, Reverse, 5´- GGTCTCTCTCTTTTTCACAAGC-3'
JAK2	Forward, 5´-TTTGAAGACAGGGACCCTACACAG-3´, Reverse, 5´-TCATAGCGGCACATCTCCACA-3′
STAT3	Forward, 5´-CACCCATAGTGAGCCCTTGGA-3´, Reverse, 5´- TGAGTGCAGTGACCAGGACAGA-3′
β-actin	Forward, 5'-GGTGAAGGTCGGTGTGAACG-3′, Reverse, 5´-CTCGCTCCTGGAAGATGGTG-3´

**Table 2:** Serum levels of AMY, ALT, and AST in different groups (U/L, n=8, x±s).

**Group**	**AMY**	**ALT**	**AST**
Control	547.35±21.48	26.51±6.35	20.18±3.24
SAP	4179.94±258.35^*^	337.56±21.63^*^	456.27±30.18^*^
SAP-AG490	3587.73±208.23^*#^	265.24±17.52^*#^	354.26±25.89^*#^

**Note: ** *P<0.05 *vs*. control group; #*P*<0.05 *vs*. SAP group

## Data Availability

The data generated in the present study may be requested from the corresponding author.

## LIST OF ABBREVIATIONS

AP: Acute pancreatitis
SAP: Severe acute pancreatitis
JAK2: Janus kinase 2
STAT3: Signal transducer and activator of transcription 3
LPS: Lipopolysaccharide
KC: Kupffer cell
AMY: Amylase
ALT: Alanine aminotransferase
AST: Aspartate aminotransferase
H&E: Hematoxylin-eosin
ELISA: Enzyme-linked immunosorbent assay
RT-qPCR: Reverse Transcription-quantitative PCR
FITC: Fluorescein isothiocyanate
BSA: Bovine serum albumin
PVDF: Polyvinylidene fluoride
HRP: Horseradish peroxidase
TLR4: Toll-like receptor 4
NF-κB: Nuclear factor kappa-B
MAPK: Mitogen-activated protein kinase
IRF3: Interferon regulatory factor 3
ICU-AW: Intensive care unit-acquired weakness
IFN-γ: Interferon-γ
IL-6R: Interleukin-6 receptor
IFN-γR: Interferon-γ receptor
NLRP3: NOD-like receptor pyrin domain-containing 3
DMSO: Dimethyl sulfoxide
DMEM: Dulbecco’s modified Eagle medium
BCA: Bicinchoninic acid
SDS-PAGE: Sodium dodecyl sulfate-polyacrylamide gel electrophoresis
SIRS: Systemic inflammatory response syndrome
